# Investigating the Consumer Choices of Gen Z: A Sustainable Food System Perspective

**DOI:** 10.3390/nu17030591

**Published:** 2025-02-06

**Authors:** Ewa Halicka, Joanna Kaczorowska, Krystyna Rejman, Marta Plichta

**Affiliations:** Institute of Human Nutrition Sciences, Warsaw University of Life Sciences WULS-SGGW, Nowoursynowska 159c, 02-776 Warsaw, Poland; joanna_kaczorowska@sggw.edu.pl (J.K.); krystyna_rejman@sggw.edu.pl (K.R.); marta_plichta@sggw.edu.pl (M.P.)

**Keywords:** food choices, generation Z, sustainable diets, young adults, food systems, Poland

## Abstract

**Background/Objectives:** Developing nutrition-oriented and more responsible consumer behaviors is crucial for the well-being of current and future generations. Food choice determinants and concerns of Gen Zs—also referred to as Post-Millennials, or Zoomers—were explored from a sustainable food system perspective to identify factors encouraging young people to be on the front line of this much-needed transformation. **Methods:** Quantitative data were collected with the use of the CAWI method from 650 adults representing Generation Z living in Polish cities. Statistical analysis identified three consumer segments, and cluster (CL) means were statistically contrasted using a one-way ANOVA with Scheffé tests for post hoc comparisons. **Results:** The research results indicate that the key considerations of food choice in the studied Gen Z sample are taste, price, and health. External factors, such as social media influencers and consumer trends, play a relatively minor role in decision-making. Gen Zs were more aware of the link between their eating patterns and health than the link between their eating habits and the natural environment. Members of the biggest cluster (CL3), constituting 48.3% of the total sample, declared the highest level of agreement with statements referring to links between diet, environment, and health from the food system perspective. This most allo-centered (community-oriented, altruistic) consumer segment, differing significantly with gender to other CLs was more concerned about climate change and protecting the natural environment and open to changing its dietary habits. **Conclusions:** Implementing research-based policy measures focusing on Generation Z, especially young women, as potential leaders and drivers of food system change is recommended.

## 1. Introduction

How food is produced, processed, distributed, and consumed is widely recognized as unsustainable in all food system domains [1,2,3,4]. A just transition towards more responsible food choices is an immediate challenge closely linked to the aspirations of the 2030 Agenda and related to the objective of achieving the United Nations (UN) Sustainable Development Goals [5,6]. Ultimately, sustainable food systems deliver food security and nutrition for all so that the economic, social, and environmental bases that will generate food security and nutrition for future generations are not compromised [7]. Recent studies show that consumer knowledge about sustainability issues influences attitudes and behaviors, enhancing perceived value and enabling informed choices [8]. Attitudes and knowledge are significant predictors of sustainable food consumption among youth [9], a cohort exposed to the negative outcomes of these systems like no other generation [9,10].

Understanding the motives behind food behaviors and choices is crucial for developing strategies to promote healthier, more sustainable diets amid growing concerns over environmental degradation, public health issues, and food security. However, more holistic research is needed on the web of factors influencing everyday decision-making processes to bridge the gap between concerns and behaviors in different populations. Youth, defined for statistical purposes by the UN as persons between the ages of 15 and 24 years [11], is viewed by many organizations as pivotal in driving more sustainable consumption patterns and mitigating the negative outcomes of food systems [12]. Globally, this demographic has the potential to play a more active role in mitigating the effects of environmental changes and climate change, which are likely to accelerate and intensify during their lifetimes [13]. Young people are also increasingly emerging as visible agents of change, especially through networks that create, transform, and distribute food systems knowledge, such as Fridays for Future and Act4change [14,15].

European countries appear to be the frontrunners in gathering scientific data about consumer knowledge and willingness to adopt sustainable, nutrition-sensitive practices [16]. However, a recent survey conducted in 11 European Union (EU) countries revealed that consumers still tend to underestimate the environmental impact of their eating habits. Factors such as price, a lack of information, and limited availability are perceived as barriers to a shift to more sustainable diets [17]. Poland, an EU member state that has experienced rapid socio-economic transitions, offers a unique context for researching food consumer behaviors from this perspective. Previous studies found considerable differences among city dwellers regarding their concerns about the environmental sustainability of diets [18]. However, more research is needed to explore this in more depth. This is important, as food environments with abundance and high availability of food products have been shown to be linked to poorer health outcomes [18,19].

Because a shift towards sustainable diets and consumer choices is necessary for humanity to prosper in the coming years and young people are on the front line of this much-needed shift [20], this study focused on two objectives:

1. Identifying the key food choice determinants and concerns of Gen Zs (also referred to as Zoomers or Post-Millennials) living in Polish cities;
2. Assessing what share of adult Gen Zs are aware of, feel responsible for, and actively discuss the interlinkages between diets, health, and the natural environment from the food system perspective.

Since digital technology has accompanied Post-Millennials since birth, today’s young adults are likely to be technology-dependent and to rely on the internet and smartphones for much of their daily communication, social interaction, education, and news [21]. Further, considering that almost all Post-Millennials have a smartphone or access to one and use their smartphones to connect to the internet our research also sought to determine if Polish young adults feel that external factors, such as social media, have an important impact on their daily food-related choices.

## 2. Materials and Methods

### 2.1. Data Collection and Sample

The primary data on food-related behaviors in the context of sustainability challenges presented in this article were collected in Poland in 2023 using the Computer-Assisted Web Interviewing (CAWI) technique. This study was conducted in accordance with the Declaration of Helsinki and was approved on 28 January 2022 by the Committee for the Ethics of Research Involving Human Participants of the Institute of Human Nutrition Sciences at Warsaw University of Life Sciences (protocol 01/2022). Informed consent was obtained from all subjects involved in this study.

An original questionnaire, designed by the authors with a focus on food consumption choices among Polish adults (in different age groups), was partially inspired by Schwartz’s values instrument [22], which was validated in the context of environmental concerns in another East European country [23]. The questionnaire was tested among a group of 14 young adults living in the Mazovia voivodship of Poland who fulfilled this study’s inclusion criteria. After introducing minor editorial changes to increase the clarity of the questions and responses, a country-wide study was conducted in May 2023 using a consumer panel of a commercial market research company comprising more than 100,000 users. Quota sampling was applied, as respondents were selected to represent adults living in cities in all 16 provinces of Poland (N = 1300). The survey’s participation criteria were the ages of the respondents and equal participation of men and women.

This article focuses on data collected from 650 young adults born between 1999 and 2005 and living in Polish cities. According to Statistics Poland, given that 60% of the country’s population lives in urban areas, the number of 18–24-year-olds living in cities is estimated to be 2.4–3.4 million [24]. City dwellers were chosen for this research based on previous observations showing that environmental challenges such as pollution, waste management, and resource depletion are more pronounced in cities and directly affect the daily lives of urban populations [25].

### 2.2. Measures and Data Analysis

The answers to the survey questions (Table A1) were analyzed using IBM SPSS Statistics version 29 (SPSS Inc., Chicago, IL, USA). In Question 1 (Q1), the respondents were asked to rate the importance of 17 pre-determined and randomly rotated factors related to food choice using a five-point Likert scale from ‘1’ (‘unimportant’) to ‘5’ (‘very important’). Thematic analysis was used to categorize these factors into individual, collective, and external subgroups. Q2 included a list of nine pre-determined and randomly rotated topics focused on food system domains, such as the natural environment, health, and social responsibility. The respondents were asked to indicate, on a five-point Likert scale ranging from ‘0’ (‘never’) to ‘4’ (‘very often’), how frequently they discuss these topics with people close to them (in their immediate environment). Q3 and Q4 were related to Gen Z’s awareness regarding diet, the natural environment, and health issues from the perspective of food system linkages and their willingness to change their dietary habits by increasing the consumption of plant foods and reducing that of animal foods.

The collected responses were interpreted to characterize the sample and describe the identified clusters. Q3 used a 5-point Likert scale to evaluate the respondents’ agreement with selected behavior-related statements. In Q5, the study participants were asked to select the correct definition of sustainable food consumption from four options or admit to being unfamiliar with it. The correct response was simplified to “when everyday food consumption is carried out to minimize the impact on the natural environment” like in previous research conducted among other populations living in Poland [25,26].

The first step of the conducted data analysis focused on identifying consumer segments (clusters) using the 17 factors related to food choice (Q1) and the frequency of discussion of nine topics related to food systems (Q2). Factor analysis was performed using the principal component analysis (PCA) method, followed by Varimax rotation with Kaiser normalization to reduce the number of variables and reveal any strong relationships between them. We found these questions particularly interesting in the context of food systems and consumer choice. In total, Q1 and Q2 contained 26 variables. An eigenvalue criterion of >1 was used to determine the number of significant components, while a Scree Plot was used for validation. This analysis identified five components explaining 57.2% of the original variance. Next, a cluster analysis (K-means method) for respondent segmentation was performed. The three-cluster solution was chosen, and the cluster means were statistically contrasted using a one-way ANOVA with Scheffé tests for post hoc comparisons.

Descriptive statistics (frequencies, means, and cross-tabulations), ANOVA, and Pearson’s Chi-square independence tests were used to examine the differences between the three clusters. The details depended on the measurement scales of the variables, and a level of *p* ≤ 0.05 was considered significant.

## 3. Results

### 3.1. Sample and Cluster Socio-Demographic Characteristics

The socio-demographic characteristics of the total sample (N = 650, 325 women and 325 men) and the three identified clusters are presented in Table 1. The average age of the sample group was 21.3 years. More than one-third (37%) of the sample lived in small cities of up to 49 thousand inhabitants, 26% lived in medium-sized cities (up to 199 thousand inhabitants), and 37% were from large cities (over 200 thousand inhabitants). Two-thirds of the respondents reported living with their parents, 26% cohabitated with people of the same age, and 8% lived alone. Almost 16% of the studied sample had children.

Although the shares of men and women in the total sample were equal, the three identified clusters varied significantly with regard to gender (*p* = 0.000). In the biggest cluster (CL3), constituting 48.3% of the total sample, more than 60% were women. In comparison, women formed minorities in CL1 (44.4%) and CL2 (37.6%).

More than half (54%) of the surveyed 18–24-year-olds had completed secondary school, 27% held a higher education degree, and 19% of them remained at the primary or vocational level. As shown in Table 1, the largest share of Gen Zs with university degrees was observed in CL3. However, the statistical differences between the clusters were not significant (*p* = 0.329) with regard to the respondents’ education levels. Similarly, the living situations of the studied Gen Zs did not significantly differ between the clusters (*p* = 0.091). However, as presented in Table 1, the largest share of young adults living with their parents or family members was observed in CL1 (nearly 74%) compared to CL2 (68.6%) and CL3 (60.5%).

Regarding the two remaining variables, city size and parenthood, the clusters differed significantly (*p* = 0.030 and *p* = 0.009, respectively). The share of Gen Zs with children was higher in CL1 (23%) compared to CL2 (10.5%) and CL3 (15.9%).

### 3.2. Factors Determining Young Adults’ Food Choices

According to the collected data, the three most important factors determining food choices in the surveyed sample of adult youth were taste (4.67 on a 5-point scale), price (4.40), and health (4.33). Another factor considered rather crucial by the respondents was convenience in meal preparation (4.05). The factors seen as least important were the opinions of influencers and bloggers (2.17), consumer trends (2.66), and advertising (2.90).

The cluster means for the factors determining food choices are presented in Table 2. Superscript letters indicate if the clusters differ significantly for each factor (*p* ≤ 0.05).

Based on a one-way ANOVA with Scheffé tests for post hoc comparisons, CL3 had the highest means. However, in the case of individual factors (price, health, taste, and convenience) and principles of healthy eating, these were rather closely mirrored by CL2.

As shown in Table 2, the importance of factors linked to five environmental sustainability challenges, such as food certification, reusable packaging, and organic production, oscillated between 3.24 and 3.71 in the overall sample. The means were highest in CL3, ranging from 3.75 to 3.98. In CL2, all of these factors, except certified quality, were rated below 2.80, with reusable packaging being the lowest rated factor (2.51).

The least essential food choice determinants for the studied sample were the external (socio-cultural) factors, especially the opinions of influencers and bloggers (2.17/5.00). As with other groups of food choice determinants, the importance of socio-cultural factors varied significantly between the clusters. CL2 had the lowest means in all cases. CL1 members paid the most attention to expert recommendations (3.67/5.00) and consumer trends (3.35/5.00). For CL3, in the context of supporting Polish producers, the Polish origin of food products was most important (4.01/5.00).

### 3.3. Understanding Sustainability Challenges from the Food System Perspective

The collected data show that the studied 18–24-year-olds living in Polish cities were definitely more in agreement with the fact that their eating patterns affect their health (4.30/5.0) than the fact that they affect the natural environment (3.62). The conducted one-way ANOVA showed statistically significant differences between the clusters. The results of Scheffé tests for post hoc comparisons are presented in Table 3.

The surveyed Zoomers agreed with most of the statements referring to the links between food system domains, specifically between eating habits and health. The highest level of agreement was noted in CL3 and CL2, with CL1 being significantly lower. CL1 and CL2 were significantly less concerned about climate change, and protecting the natural environment was also of lower interest to them than CL3.

Overall, as can be seen in Table 3, CL2 had the lowest levels of agreement with all statements in Q3, which indicates that CL2 members were least aware of the links between food system components and least concerned with the sustainability of their behaviors.

Based on the respondents’ answers to Q4, 36% of the total sample had not considered changing their nutritional habits, 37% had contemplated making sustainability-oriented changes, and 27% had already increased their plant food consumption and reduced their animal food intake. Statistical differences were found between the clusters, with CL2 being the subgroup that was least open to change, as 57% of the CL2 members declared that they had no intention of changing their diets and making them more sustainable.

Our results also show that almost one-third (32%) of the Gen Z sample correctly indicated that sustainable food consumption implies that “everyday nutrition is carried out in such a way as to minimize its environmental impact”. However, a similar share of the respondents (33%) chose one of the incorrect definitions, stating that “food consumption is sustainable when the energy value of consumed food equals the body’s energy expenditure”. A smaller share (17%) of the research participants wrongly believed that food consumption is sustainable when “the share of plant and animal products in the food consumed is the same”. The option stating that sustainable food consumption means that “the cost of food is adjusted to the financial capacity of the household” was chosen by 9% of the studied Gen Zs. An identical share (9%) admitted they were unfamiliar with “sustainable food consumption”.

### 3.4. Discussing Food, Health, and the Natural Environment

Based on the responses to Q2, the most frequently discussed topic among the surveyed Gen Zs (out of the nine pre-determined issues) was the link between diet and health. This was followed by food waste and the seasonality of food products. The frequency means for these three topics were highest in CL3, surpassing 3.60 on a scale from 0 to 4. A one-way ANOVA showed statistically significant differences between the clusters (*p* ≤ 0.001). The results of Scheffé tests for post hoc comparisons are shown in Table 4.

Reducing purchases of plastic-bottled beverages (incl. water) and buying foods produced locally were also discussed often among the interviewed young consumers. In comparison, food miles were discussed less frequently (2.46/4.00 in the total sample). In all cases, the CL2 members admitted to talking about the environment and food-related issues less frequently.

Overall, as shown in Table 4, CL2 had significantly lower means compared to the other consumer segments. Only in the case of the two most discussed topics—the influence of diet on health and food waste—did CL2 members respond similarly to CL1. Limiting the consumption of meat and meat products was discussed more often in CL1 and CL3 compared to CL2.

## 4. Discussion

### 4.1. Dominance of Taste, Price, and Health

Our research findings show that food choice determinants among Gen Zs in Poland are multifaceted and involve many individual, environmental, and external factors. The key considerations—taste (4.67/5.00), price (4.40/5.00), and health (4.33/5.00)—align with those identified in other studies, underlining their dominant role in shaping the dietary preferences and purchasing decisions of consumers in the EU. A review showed that people in highly developed countries use various individual coping strategies in community and consumer nutrition environments to make optimal purchasing decisions, often within financial constraints [27].

In the case of food products, taste is a deeply ingrained factor in dietary behaviors, with individuals often prioritizing immediate gratification over long-term considerations. According to recent research in Austria [28], the place of origin and price are the most important attributes of ‘plant-based burger patties’ for individuals aged ≤29 years. In Finland, the perception of high prices for plant-based foods among university students was the most relevant and significant barrier to climate-friendly food choices [29]. Our results also resonate with the findings of another Polish study, which highlighted the importance of sensory appeal and cost-effectiveness in food decisions among young consumers [30]. However, research conducted among parents of school-aged children in urban Poland demonstrated that price was a less important factor (3.92/5.00) when buying food for their children [31]. This suggests a shift in priorities once individuals take on caretaking responsibilities.

Additionally, as individuals become more secure economically, the price of food may become a less important factor in purchasing choices. This reflects many young adults’ economic constraints, particularly those of students and early-career professionals, and is consistent with findings that price is often seen as a barrier to adopting more sustainable diets [32,33]. A survey of 1000 US Gen Zs [34] also revealed that taste, price, and convenience emerged as the most important factors motivating food choices. More than half of the sample indicated that the most important issue the food industry should address is the cost of food. Thus, they confirmed the existence of a price barrier in food choices. The importance of price in young adults’ food choices was also pointed out in British FSA (Food Standard Agency) survey results [35] covering all age groups in the population from 16 to 66+ years. Gen Zs and 46–55-year-olds reported in the highest proportions (21 and 22%, respectively) that they make their food choices mainly based on price. Gen Z young adults ranked price in their top three factors influencing food decisions in the highest proportion (58%). According to the authors, the survey results suggest this is a life-stage effect, as price becomes less important with age. The respondents in the studied sample of Gen Zs indicated their own health as the third food-choice factor. Their motivation for choosing health-promoting foods was also evidenced by the high scores (over 3.50) given for four other choice determinants, i.e., healthy eating principles, high quality confirmed by certificates or symbols, and recommendations of healthy lifestyle specialists. Furthermore, their health concern was confirmed by the overall consensus that “eating habits affect health”, which received the highest score (4.30) among statements covering all domains of a sustainable food system.

Due to the perception of health in food choices, our results align with the characteristics of the young adult generation globally. According to the 2021 NielsenIQ Global Health and Wellness Report [36], there has been a huge shift in ideas about health and well-being since the pandemic. Well-being is a far more comprehensive term than health or wellness, as it considers a broader universe of personal factors and speaks to the goals of a well-rounded life. Younger consumers were much more interested in foods they perceived as healthy than older consumers. Representatives of Gen Z (the report assumed their age to be under 20 years) were the most health-oriented population. More than 40% said they would be happy to pay a higher price for ‘healthier’ products (compared to around 30% of Millennials and 20% of Generation BB (Baby Boomers)). It is worth noting that the authors of the FSA report [35] were more skeptical about the role of health in young consumers’ food choices. Stereotypes about Gen Z are rarely supported by evidence, and solid data are lacking. In their view, it is widely reported that Generation Z is leading the vegan and vegetarian movement, but this does not seem to have translated into their consumption behavior. Their findings show that 16–24-year-olds are least likely to say they eat fruits and vegetables, while they consume more fast food and takeaway meals. However, evidence suggests that this is due to their life stage rather than a cohort effect. Healthier food choices increase with age as people become more health-conscious and obtain higher incomes. The lack of longitudinal data examining food consumption makes it difficult to assess whether these are true generational differences.

### 4.2. Environmental Concerns

Our findings demonstrate that overall Gen Zs in Poland consider environment-related factors less critical than individual/personal determinants. The surveyed young adults were more aware of the link between their eating habits and health (4.30 on a scale of 1–5) than the link between their eating habits and the natural environment (3.62/5.00). Among the factors related to environmental sustainability, “high quality confirmed by certificates or symbols” was perceived as the most important determinant of choice (3.71/5.00).

Although personal motives can influence the buying decisions of Gen Zs, who are often price-sensitive, Post-Millennials also display a high awareness of environmental conservation issues [37,38]. The so-called universalism values, which encompass caring for nature, the environment, and other people, can already be detected in adolescents from different cultures and distinguished from their other values [22]. Therefore, research-based education programs implemented in schools, which include topics on planetary diets, plant sources of protein, reducing food waste, etc., can further increase youth’s sustainability awareness. A 2020 study conducted in Poland among Gen Zs living in cities noted that respondents cared for the natural and social environment by segregating trash, saving water, and turning off lights. Their behaviors aimed to reduce costs or resulted from legal requirements [39].

In our study, 36% of the surveyed Gen Zs declared that they had not considered changing their nutritional habits, 37% contemplated making such changes, and 27% had already increased their plant food consumption and reduced their animal food intake. Research shows a strong attachment to meat, which is a predictor of resistance (low level of willingness) to reducing meat consumption, among people living in Polish cities [31]. Different levels of meat attachment factors drive consumers who intend to or have already reduced their meat consumption compared to those with no willingness to change; however, more research is needed exploring the determinants of change and possibilities for effective communication about meat reduction on an individual level in different cultural settings.

According to a 2019 study [17], on average, only slightly over 10% of those surveyed in 11 EU states agreed that what they eat harms the environment, whereas 63.6% disagreed (ranging from 54.6% in Belgium to 71.2% in Greece). The environmental dimension was most likely to come spontaneously to the studied consumers’ minds when they thought of “sustainable food”. The majority of them cited a “low environmental impact”, the “use of pesticides/GMOs to be avoided”, and “local supply chains” (rightly or wrongly, it is a general perception that locally produced food has a lower environmental impact). By no means can it be deduced that consumers give little consideration to other elements of sustainability (e.g., health and animal welfare), but they are less likely to spontaneously associate these aspects with the term “sustainability”.

A recently conducted scoping review showed that consumers generally lack knowledge about food system sustainability and, in the main, are currently unwilling to adopt a sustainable diet. Misconceptions surrounding sustainable diets exist, perhaps due to a lack of information in this area [16]. Actions aimed at enabling youth to be active participants within food systems by increasing their food literacy were shown to support better food choices throughout life [40]. Many countries implement education programs in school settings to improve food-related decisions. Such tools, which also promote food literacy, should be considered part of the multifaceted actions needed to achieve healthy and sustainable eating habits [41,42].

### 4.3. External Influences, Including Social Media

In light of our study, adult youth living in Poland (especially CL2, 62% of whom were men) do not perceive external factors linked to social media, such as consumer trends or the opinions of influencers and bloggers, as important in their food-related purchasing decisions (2.66/5.00 and 2.17/5.00, where ‘2’ indicates ‘rather unimportant’). This finding may suggest that overall increased access to information through digital platforms, although potentially allowing consumers to make more informed decisions, is not perceived by them as an important determinant of food choice. The only socio-cultural factor the studied Gen Zs considered important (CL3 members considered this factor more important than CL1 and CL2 members) was the local origin of products, supporting local Polish producers.

These results shed new light on the role of social media in influencing young people’s consumer behaviors, incl. diets. It must be noted that social media is a broad term encompassing websites and applications utilized for various purposes, such as communicating with friends and family, gathering information, staying updated with current trends, entertainment, education, and more [43]. Gen Zs, who are often called digital natives because they grew up surrounded by technology, including the internet, smartphones, and social media, are exceptionally skilled at navigating digital tools and platforms [21].

The use of new technologies was evaluated among young people living in Spain in the context of sustainable consumption education [44]. It was shown that among young people, purchasing, using, and managing new technologies and the virtual world may be a good entryway for reflecting on healthier, more sustainable, and more social lifestyles. Research on Polish Zoomers’ perceptions of the role of digital apps in increasing environmental sustainability awareness proved that food-saving applications such as TooGoodtoGo may be important in creating pro-ecological behavior. However, to be effective, such apps must meet users’ expectations in terms of, among others, safety and the costs of use [39]. To increase Generation Z’s recognition of pro-environmental mobile applications, actions should be taken to promote environmentally and socially sustainable attitudes [45]. Another study showed that young adults see some motivational aspects of social media usage. However, most demonstrated a passive attitude and infrequent activity on such websites. Few students participating in the study published actively online, as they expected valuable content but were not inclined to disseminate it [46].

### 4.4. Consumer Segmentation of Surveyed Young Adults

Among the three distinct consumer groups identified in the surveyed Gen Z sample based on food choice determinants and the frequencies of discussions around food system issues, one group (CL3) seemed most involved. CL3 members, constituting almost half of the sample, had the best sustainability knowledge and engaged in discussions on food system topics most frequently. They were also more open to changing their own nutritional behaviors by increasing plant food consumption and reducing meat intake. Data analysis showed that this consumer segment was characterized by a higher share of women (60.5%) compared to CL1 and CL2 (44.4% and 37.6%, respectively). This demographic characteristic supports the results of other research, which revealed the existence of a psychological connection between sustainability and femininity and pointed out that men tend to avoid eco-friendly behaviors and lifestyles because they are judged as feminine [47]. Women also have significantly more positive attitudes towards vegetarian and vegan diets [48]. Interestingly, the CL3 members, who were more educated than the respondents from the other clusters, declared the highest agreement with statements confirming their awareness of links between diets, health, and the natural environment. This may also suggest that education encourages young adults to discuss food systems and may lead to adopting more sustainable practices.

CL1 can be described as the most “self-centered” group, caring primarily for personal resources. This may be linked to the fact that CL1 included a larger share of parents. In comparison, CL2, whose choices were influenced by individual factors to a degree similar to CL3 but were significantly less influenced by socio-cultural determinants, can be referred to as the more pleasure-seeking (“idiocentric” or “hedonistic”) consumer segment.

Although individual factors were the most important determinants of food choice in all clusters, the members of the largest consumer segment (CL3) were significantly more aware and knowledgeable about issues related to sustainable consumption principles. It can be assumed that their mindset is more system-oriented. Therefore, CL3 can be described as “system/community-oriented” or “allo-centered”, referring to the term “allocentric”, which can be defined as giving priority to the collective self. Regarding values, CL3 showed the strongest universalism (encompassing caring for nature (biospheric) and caring for people (altruistic)) values. A longitudinal study conducted in Southern Italy, which focused on the changes in values in adolescence, demonstrated that universalism values are less prioritized in youth than in later life [49].

In light of our study, it can be concluded that food choice determinants and food system concerns among young adults living in Polish cities are multifaceted and involve an array of individual, environmental, and external factors. We recommend that future research on this demographic should focus on young women as potential leaders and drivers of change. Accelerating shifts towards sustainability and healthier outcomes require Gen Zs to feel more aware of the impacts of their behaviors and choices on the environment and society. Food policy measures, including communication campaigns to empower Gen Zs to make sustainable and nutrition-oriented choices, should emphasize that sustainable healthy diets can also be tasty and easy to prepare.

## 5. Limitations and Future Research

While this study provides valuable insight into the food choices of young adults in Poland, it is not without limitations that should be addressed in future research. Firstly, this study was limited to city residents, and rural consumers, who may face different environmental, economic, and cultural influences, were not included. As food choice determinants may differ between urban and rural settings, future studies should consider including both urban and rural groups for a more comprehensive understanding of this topic.

The current study also has several limitations that arose from the attitude–behavior gap, which is typical for consumer studies. Even though the survey was conducted anonymously, the answers could have been influenced by the matter of the research itself. It can be assumed that knowledge about sustainable food choices is limited; therefore, it entails a narrow spectrum of topics raised by youth.

One of the potential limitations of this study is that although food sustainability has many dimensions, including economic, environmental, and social dimensions (also described as the profits, planet, and people pillars), the survey questions focused mainly on the health and environmental domains. The number of survey questions was limited, as young people are often easily discouraged while filling in long online questionnaires. To provide a deeper understanding of the complexities of food consumption choices, qualitative data should also be collected to further illuminate opportunities to involve youth in shifting food systems towards sustainability.

## Figures and Tables

**Table 1:** Socio-demographic characteristics of the sample (total and clusters, in %).

Variable		Total Sample	Clusters *		
			1	2	3
		N = 650	N = 126	N = 210	N = 314
Gender	Female	50.0	44.4	37.6	60.5
	Male	50.0	55.6	62.4	39.5
Age	Average (years)	21.3	21.3	21.0	21.5
Education	Primary or vocational	19.2	19.8	21.4	17.5
	Secondary	53.5	57.1	54.3	51.6
	Higher	27.2	23.0	24.3	30.9
City size	Small	37.4	41.3	38.1	35.4
	Medium	25.8	32.5	20.5	26.8
	Large	36.8	26.2	41.4	37.9
Living with	Family/parents	65.7	73.8	68.6	60.5
	No one (alone)	7.8	7.1	8.6	7.6
	Peers	25.5	19.0	21.9	30.6
	Refusal to answer	0.9	0.0	1.0	1.3
Children	Yes	15.5	23.0	10.5	15.9

* Clusters differed significantly only with regard to gender (*p* = 0.000), city size (*p* = 0.030), and parenthood (*p* = 0.009).

**Table 2:** Post hoc analysis of the means of the total sample and the clusters for factors determining food choices among adult youth.

Factors *	Total Sample	Clusters		
		1	2	3
I. Individual				
Taste	4.67	3.82 ^a^	4.84 ^b^	4.89 ^b^
Price	4.40	3.63 ^a^	4.51 ^b^	4.63 ^b^
My health	4.33	4.04 ^a^	4.42 ^b^	4.39 ^b^
Convenience of meal preparation	4.05	3.61 ^a^	3.91 ^b^	4.32 ^c^
Principles of healthy eating	3.91	3.69 ^a^	3.88 ^a,b^	4.02 ^b^
II. Environmental				
High quality confirmed by certificates or symbols	3.71	3.59 ^a^	3.39 ^a^	3.98 ^b^
Impact on the natural environment	3.50	3.65 ^b^	2.71 ^a^	3.97 ^c^
Local/Polish origin of food to reduce transport	3.49	3.68 ^b^	2.73 ^a^	3.92 ^c^
Reusable or recyclable packaging	3.37	3.53 ^b^	2.51 ^a^	3.87 ^c^
Organic production	3.32	3.46 ^b^	2.60 ^a^	3.75 ^c^
III. Socio-cultural (external)				
Recommendations of healthy lifestyle specialists	3.56	3.74 ^b^	3.46 ^a^	3.56 ^a,b^
Local origin to support Polish producers	3.54	3.52 ^b^	2.86 ^a^	4.01 ^c^
Opinions of peers and acquaintances	3.35	3.40 ^a,b^	3.15 ^a^	3.47 ^b^
Recommendations of research institutes or experts	3.24	3.67 ^c^	2.72 ^a^	3.40 ^b^
Advertising and other promotions	2.90	3.24 ^b^	2.67 ^a^	2.92 ^a^
Consumer trends	2.66	3.35 ^c^	2.19 ^a^	2.69 ^b^
Opinions of influencers and bloggers	2.17	3.06 ^b^	1.86 ^a^	2.02 ^a^

* On a scale from 1 to 5, the respondents rated the factors as 1—unimportant, 2—rather unimportant, 3—neither important nor unimportant, 4—rather important, or 5—very important. The superscript letters indicate whether the cluster means differ significantly (*p* ≤ 0.05). If the cluster means have the same superscript letter, they do not differ statistically.

**Table 3:** The respondents’ levels of agreement with statements linking diets, the natural environment, and food system health outcomes.

Statements	Total Sample	Clusters		
		1	2	3
My eating habits affect my health.	4.30	3.70 ^a^	4.43 ^b^	4.46 ^b^
I am concerned about climate change.	3.82	3.58 ^a^	3.42 ^a^	4.18 ^b^
Environmental pollution impacts my daily life.	3.82	3.73 ^b^	3.41 ^a^	4.13 ^c^
Protecting the natural environment is important to me.	3.77	3.55 ^a^	3.39 ^a^	4.11 ^b^
I feel responsible for ensuring our planet will secure the needs of future generations.	3.68	3.60 ^b^	3.21 ^a^	4.02 ^c^
My eating habits affect the natural environment.	3.62	3.54 ^b^	3.21 ^a^	3.96 ^c^

On a scale from 1 to 5 (1—definitely not; 2—rather not; 3—neither yes nor no; 4—rather yes; 5—definitely yes). The superscript letters indicate whether the cluster means differ significantly (*p* ≤ 0.05). If the cluster means have the same superscript letter, they do not differ statistically.

**Table 4:** Frequency of discussing sustainable food system-related topics among respondents.

Topics	Total Sample	Clusters		
		1	2	3
Influence of diet on health	3.60	3.40 ^a^	3.37 ^a^	3.83 ^b^
Food waste	3.44	3.20 ^a^	2.99 ^a^	3.84 ^b^
Seasonality of food products	3.23	3.29 ^b^	2.54 ^a^	3.67 ^c^
Reducing purchases of water and other drinks in plastic bottles	3.02	3.28 ^b^	2.30 ^a^	3.41 ^b^
Buying local foods	3.00	3.37 ^b^	2.17 ^a^	3.42 ^b^
Environment-friendly food production	2.87	3.26 ^b^	2.10 ^a^	3.23 ^b^
Links between food consumption and the natural environment	2.80	3.36 ^c^	2.09 ^a^	3.05 ^b^
Limiting the consumption of meat and meat products	2.76	3.20 ^b^	2.16 ^a^	2.99 ^b^
Shortening the distances in food transport (food miles)	2.46	3.25 ^c^	1.74 ^a^	2.62 ^b^

On a scale from 0 to 4 (0—never, 1—seldom, 2—sometimes, 3—often, 4—very often). The superscript letters indicate if the cluster means differ significantly (*p* ≤ 0.05). If the cluster means have the same superscript letter, they do not differ statistically.

## Data Availability

The data presented in this study are available on request from the corresponding author.

## Appendix A

**Table A1:** List of questions used in Gen Z study (2023).

Q1.	How important for you are the following factors when choosing food? Price; My health; Taste; Convenience of meal preparation; Principles of healthy eating: Impact on the natural environment; Organic production; Reusable or recyclable packaging; High quality, confirmed by certificates or symbols; Local/Polish origin of foods to reduce transport; Recommendations of research institutes or experts; Advertising and other promotions; Consumer trends; Local/Polish origin of foods to support Polish producers; Opinions of influencers and bloggers; Opinions of peers and acquaintances; Recommendations of healthy lifestyle specialists (dietitians, doctors, PA trainers).
A1.	□ unimportant (1) □ rather unimportant (2) □ neither important nor unimportant (3) □ rather important (4) □ very important (5)
Q2.	How often do you discuss the following topics with people close to you? Food waste; Links between food consumption and the natural environment; Influence of diet on health; Seasonality of food products; Environment-friendly food production; Limiting the consumption of meat and meat products; Reducing purchases of water and other drinks in plastic bottles; Shortening the distances in food transport (food miles); Buying local foods.
A2.	□ never (0) □ seldom (1) □ sometimes (2) □ often (3) □ very often (4)
Q3.	Do you agree with the following statements? Protecting the natural environment is important to me; Environmental pollution impacts my daily life; I am concerned about climate change; My eating habits affect the natural environment; My eating habits influence my health; I feel responsible for ensuring our planet will secure the needs of future generations.
A3.	□ Definitely not (1) □ Rather not (2) □ Neither yes, nor no (3) □ Rather yes (4) □ Definitely yes (5)
Q4.	Do you see the need to change your nutritional habits by increasing the consumption of plant foods and reducing the consumption of animal-derived products?
A4.	□ No, I have not thought about such changes at all □ Yes, I have made such changes already □ Yes, I am thinking of making such changes
Q5.	What does the term “sustainable food consumption” mean?
A5.	□ the energy value of consumed food equals the body’s energy expenditure □ everyday nutrition is carried out in such a way as to minimize its environmental impact □ the share of plant and animal products in the food consumed is the same; □ the cost of food is adjusted to the financial capacity of the household □ I am not familiar with the term
